# Susceptibility of cervical cancer to dihydroartemisinin-induced ferritinophagy-dependent ferroptosis

**DOI:** 10.3389/fmolb.2023.1156062

**Published:** 2023-03-31

**Authors:** Hanqiang Shi, Lie Xiong, Guang Yan, Shuqin Du, Jie Liu, Yanbo Shi

**Affiliations:** ^1^ Central Laboratory of Molecular Medicine Research Center, Jiaxing Traditional Chinese Medicine Hospital Affiliated to Zhejiang Chinese Medical University, Jiaxing, China; ^2^ Jiaxing Key Laboratory of Diabetic Angiopathy Research, Jiaxing, China; ^3^ Department of Urology, Nanfang Hospital, Southern Medical University, Guangzhou, China; ^4^ School of Pharmacy, Zhejiang University of Technology, Hangzhou, China; ^5^ Oncology Department, Jiaxing Traditional Chinese Medicine Hospital Affiliated to Zhejiang Chinese Medical University, Jiaxing, China

**Keywords:** cervical cancer, dihydroartemisinin, ferroptosis, ferritinophagy, heme oxygenase-1, doxorubicin

## Abstract

The clinical therapeutics of cervical cancer is limited due to the drug resistance and metastasis of tumor. As a novel target for antitumor therapy, ferroptosis is deemed to be more susceptible for those cancer cells with resistance to apoptosis and chemotherapy. Dihydroartemisinin (DHA), the primary active metabolites of artemisinin and its derivatives, has exhibited a variety of anticancer properties with low toxicity. However, the role of DHA and ferroptosis in cervical cancer remained unclear. Here, we showed that DHA could time-dependently and dose-dependently inhibit the proliferation of cervical cancer cells, which could be alleviated by the inhibitors of ferroptosis rather than apoptosis. Further investigation confirmed that DHA treatment initiated ferroptosis, as evidenced by the accumulation of reactive oxygen species (ROS), malondialdehyde (MDA) and liquid peroxidation (LPO) levels and simultaneously depletion of glutathione peroxidase 4 (GPX4) and glutathione (GSH). Moreover, nuclear receptor coactivator 4 (NCOA4)-mediated ferritinophagy was also induced by DHA leading to subsequent increases of intracellular labile iron pool (LIP), exacerbated the Fenton reaction resulting in excessive ROS production, and enhanced cervical cancer ferroptosis. Among them, we unexpectedly found that heme oxygenase-1 (HO-1) played an antioxidant role in DHA-induced cell death. In addition, the results of synergy analysis showed that the combination of DHA and doxorubicin (DOX) emerged a highly synergistic lethal effect for cervical cancer cells, which was related also to ferroptosis. Overall, our data revealed the molecular mechanisms that DHA triggered ferritinophagy-dependent ferroptosis and sensitized to DOX in cervical cancer, which may provide novel avenues for future therapy development.

## Graphical Abstract

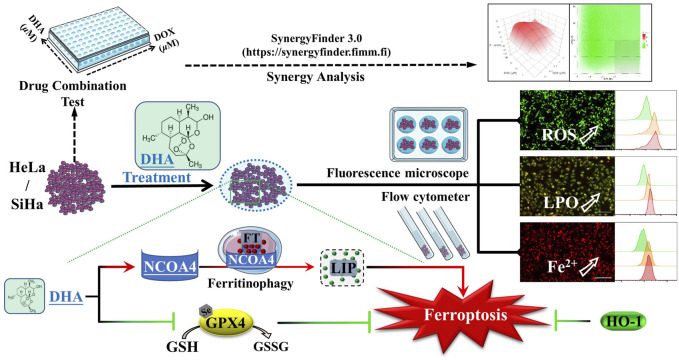

## 1 Introduction

Cervical cancer is one of the most common and lethal gynecological malignancy, affecting millions of women worldwide (Arbyn et al., 2020). Despite the vigorous promotion of HPV vaccine and the continuous improvement of screening technology, it is still the leading cause of death in women in underdeveloped countries or areas (Sung et al., 2021). And because of China’s large population, women’s death from cervical cancer accounts for 12% of women’s death worldwide. Even more alarming, its incidence rate is rising, and the incidence age is getting younger (Zhou et al., 2016; Xia et al., 2018). At present, the combined chemotherapy with targeted therapy is a standard treatment for cervical cancer, which significantly improves the survival of patients compared with chemotherapy alone (Hill, 2020). So far, the clinical outcomes of cervical cancer patients in early stages have significantly improved by standard treatments. However, due to the drug resistance and metastasis of tumor, those patients at an advanced stage or recurrent cervical cancer have a poor prognosis with limited treatment options and the therapy remains far from satisfactory (Abu-Rustum et al., 2020; Zhang et al., 2021). As a result, nowadays, the demand for new therapies with more secure, effective and feasible is becoming more and more urgent.

Ferroptosis is a new form of programmed cell death that characterized by an iron-dependent accumulation of lethal lipid peroxidation (LPO) (Dixon et al., 2012; Stockwell, 2022). And it is different from apoptosis, necrosis, and autophagy at morphological, biochemical, and genetical levels. From the perspective of antioxidant, ferroptosis is caused by a redox imbalance between the production of oxidants and antioxidants, which is driven by the abnormal expression and activity of multiple redox-active enzymes that produce or detoxify free radicals and lipid oxidation products (Tang et al., 2021a; Liu and Gu, 2022). Emerging evidences have shown that cancer cells are sensitive to ferroptosis and targeting ferroptosis has great potential to be an effective strategy and approach for cancer therapy (Hassannia et al., 2019; Chen et al., 2022). It has been reported that ferroptosis inducer RAS-selective lethal small molecule 3 (RSL3) could enhance the antitumor effect of cisplatin *via* the inhibition of glutathione peroxidase 4 (GPX4) (Zhang et al., 2020a). Noteworthy, activation of certain autophagy pathways can promote ferroptosis, which is called autophagy-dependent ferroptotic cell death, such as nuclear receptor coactivator 4 (NCOA4)-facilitated ferritinophagy, beclin 1 (BECN1)-mediated glutamate antiporter (system Xc^−^) inhibition (Zhou et al., 2020). In cervical cancer cells, the anti-cancer drug sorafenib has been shown to induce autophagy-dependent ferroptosis through the Cdc25A/PKM2/ErbB2 axis (Wang et al., 2021a). It has also been found that non-coding RNAs can affect the occurrence of ferroptosis in cervical cancer cells (Wu et al., 2021; Liu et al., 2022). And the bioinformatics analysis about ferroptosis-related genes suggests that targeting ferroptosis may represent a promising approach for the treatment in cervical cancer (Qi et al., 2021). Although the roles of ferroptosis in cervical cancer are remain rarely explored and unclear, it may be a potential therapeutic direction.

Dihydroartemisinin (DHA), first-generation derivative of artemisinin and also the primary active metabolites of artemisinin and its derivatives, is an effective antimalarial drug with low toxicity (Yu et al., 2021). Beyond the widely acknowledged anti-malarial effect, DHA has shown a variety of anticancer properties including apoptosis, autophagy and ferroptosis in different cancers, as well as enhances the efficacy of chemotherapy and targeted therapy (Dai et al., 2021; Li et al., 2021). Previous studies have confirmed that DHA has a potent lethal effect in cervical cancer cells (Lu et al., 2020), and can sensitize HeLa cells to doxorubicin (DOX) -induced apoptosis (Tai et al., 2016). Meanwhile, a clinical study also found that Artenimol-R (the succinate ester of DHA) treatment in patients with advanced cervical cancer showed an improvement of the clinical symptoms and a good tolerability (Jansen et al., 2011). Nevertheless, there is little literature to confirm the existence and importance of ferroptosis in the DHA-induced cervical cancer cell death. Thus, in the present study, we investigated the effect and mechanism of DHA on the proliferation and ferroptosis of cervical cancer cells, as well as the sensitization effect on DOX.

## 2 Materials and methods

### 2.1 Reagent and antibodies

Dihydroartemisinin (DHA, D140839, purity: ≥98%), hemin (H140872) and chloroquine (CQ, C193834) were purchased from Aladdin (China). Dimethyl sulfoxide (DMSO, A503039) and ferric ammonium citrate (FAC, A500061) were got from Sangon Biotech (China). Deferoxamine (DFO, D9533) and protoporphyrin IX zinc(II) (ZnPPIX, 282820) were purchased from Sigma-Aldrich (United States). Z-VAD-FMK (HY-16658B), ferrostatin-1 (Fer-1, HY-100579) and doxorubicin hydrochloride (DOX, HY-15142) were purchased from MedChemExpress (United States). And the antibody to ACSL4 (ab155282), GPX4 (ab125066), xCT (ab175186), HO-1 (ab82585), TfR1 (ab84036), FTH1 (ab65080) and NCOA4 (ab86707) were all purchased from Abcam (United Kingdom). Anti-β-actin and goat anti-rabbit IgG H&L were purchased from Bioker (China).

### 2.2 Cell culture and drug configuration

Human cervical cancer cells HeLa (adenocarcinoma) and SiHa (squamous cell carcinoma) were purchased from National Infrastructure of Cell Line Resource (NICR, China) and then were maintained in RPMI-1640 and MEM medium (Gibco, United States), respectively, supplemented with 10% FBS (Gibco, United States) and 100 IU/mL penicillin and 100 mug/mL streptomycin (Sangon, China) at a 37°C incubator with 5% CO_2_. DHA was prepared as a DMSO stocking solution with a concentration of 200 mM. After sub packaging, it was frozen at −20°C.

### 2.3 Cell viability assay

DHA cytotoxicity was detected by the Cell Counting Kit-8 (CCK-8; BBI, China). 5 × 10^3^ cells were seeded into each well of the 96-well plate. Then various concentrations of DHA (0, 5, 10, 20, 40 and 80 *μ*M) were added to the plates and followed by another 24, or 48-h incubation in a 37°C, 5% CO_2_ incubator. Then 10 *μ*L CCK-8 solution was added to each well and further 1 h’s incubation was carried out. The optical density (OD) was measured at 450 nm, and then the cell viability was calculated.

### 2.4 Determination of intracellular reactive oxygen species (ROS)

The fluorescent probe DCFH-DA (Sigma-Aldrich, United States) was used to evaluate the intracellular ROS levels. The methods used are according to the manufacturer’s instructions. Cells were inoculated into 6-well plates at 2 × 10^5^ cells/well and grown overnight, then incubated with different concentration of DHA for 24 h. Thereafter, the cells were stained with DCFH-DA probe at 37°C for 30 min in the dark. After washing with serum-free medium for three times, the fluorescence of cells was photographed under fluorescence inverted microscope Axio Observer D1 (ZEISS, Germany) and detected by the flow cytometer BD FACS Canto II (BD Biosciences, United States).

### 2.5 Detection of intracellular malondialdehyde (MDA)

Intracellular MDA levels in the cells were measured using micro MDA assay kit (Solarbio, China) following the instruction by the manufacturer. Briefly, the cells of each group were collected and disrupted by an ultrasonic cell pulverize. The cell suspension was centrifuged and then 100 *μ*L sample was added for the measurement, followed by the addition of 400 *μ*L of the MDA test solution. After mixing and reacting in a 100°C-water bath for 30 min, the mixture was cooled to room temperature and centrifuged. Next the supernatant was taken out and measured absorbance at 450, 532, and 600 nm wavelength with a full-wavelength microplate reader (Thermo, United States).

### 2.6 Redox status determination

2 × 10^5^ cells were seeded into each well of the 6-well plate and intervened with 0, 40 and 80 *μ*M DHA for 24 h. To assess the status of antioxidant, the collected cells were measured using the commercial assay kits of superoxide dismutase (SOD; Sangon Biotech, China), catalase (CAT; Sangon Biotech, China), reduced glutathione (GSH; Jiancheng, China) and glutathione peroxidase (GPx; Beyotime, China) strictly following the manufacturer’s instructions.

### 2.7 Lipid peroxidation (LPO) assay

LPO was investigated by BODIPY^™^ 581/591 C11 dye (Thermo, United States), which shifts fluorescence properties from red signals to green signals upon oxidation (Yi et al., 2022). Briefly, cells were seeded in 6-well plates at 2 × 10^5^ cells/well and grown overnight. After treatments, cells were loaded with 2.5 *μ*M BODIPY^™^ 581/591 C11 at 37°C for 30 min in the dark. After washing with serum-free medium for three times, the fluorescence of cells was photographed under fluorescence inverted microscope Axio Observer D1 (ZEISS, Germany) and detected by the flow cytometer BD FACS Canto II (BD Biosciences, United States).

### 2.8 RNA extraction and quantitative PCR (qPCR)

RNA was extracted and quantified according to the previous operation methods of our research group (Du et al., 2022). Total RNA was extracted from cells using TRIzol reagent (Takara, Japan) according to the manufacturer’s introduction and then converted to cDNA using a PrimeScript^™^ RT reagent kit (Takara, Japan; 37°C for 15 min, 85°C for 5 s). The qPCR assay was performed with TB Green^®^ Premix Ex Taq^™^ II (Takara, Japan; 95°C for 30 s, 1 cycle; 95°C for 5 s, 60°C for 34 s, 40 cycles) on the 7,500 Real-Time PCR system (Applied Biosystems, United States), and *β-actin* was used as an internal control. The primer sequences were listed in Table 1 and the relative expression levels were determined using the 2^−ΔΔCt^ method.

**TABLE 1:** The primer sequences.

Target gene		Primer sequence (5′to 3′)		Size (bp)
Name	Id	Forward	Reverse	
*ACSL4*	2,182	ATA​CCT​GGA​CTG​GGA​CCG​AA	TGC​TGG​ACT​GGT​CAG​AGA​GT	145
*TfR1*	7,037	TGG​AGA​CTT​TGG​ATC​GGT​TGG​TG	CAG​TGG​CTG​GCA​GAA​ACC​TTG	138
*NCOA4*	8031	GGG​CAA​CCT​CAG​CCA​GTT​AT	CAA​ACT​GCA​GGG​AGG​CCA​TA	139
*FTH1*	2,495	CCA​GAA​CTA​CCA​CCA​GGA​CTC	CAA​AGC​CAC​ATC​ATC​GCG​G	118
*GPX4*	2,879	CAG​TGA​GGC​AAG​ACC​GAA​GT	CCG​AAC​TGG​TTA​CAC​GGG​AA	104
*xCT*	23657	CAT​CTC​TCC​TAA​GGG​CGT​GC	TAG​TGA​CAG​GAC​CCC​ACA​CA	85
*H O -1*	3162	CCG​CAT​GAA​CTC​CCT​GGA​GAT​G	CTG​GAT​GTT​GAG​CAG​GAA​CGC​AG	85
*β-actin*	60	CCTGGCACCCAGCACAAT	GGGCCGGACTCGTCATAC	114

*ACSL4*: acyl-CoA, synthetase long-chain family member 4; *NCOA4*: nuclear receptor coactivator 4; *FTH1*: ferritin heavy chain 1; *GPX4*: glutathione peroxidase 4; *H O -1*: heme oxygenase 1; *xCT*: cystine-glutamate antiporter; *TfR1*: transferrin receptor 1.

### 2.9 Western blotting analysis

The cells were harvested and whole cell lysates were extracted with RIPA buffer (Solarbio, China) supplemented with protease inhibitor. Protein concentrations were determined using the BCA protein assay (Sangon, China). After quantification, equal amounts of proteins were subjected to SDS-polyacrylamide gel electrophoresis and transferred to the nitrocellulose membrane. Blocking with 5% non-fat dry milk at room temperature for 1 h, then the membrane was incubated with primary (4°C overnight) and secondary antibodies (37°C for 1 h). Then protein blots were incubated with ECL luminescence reagent (Sangon, China) and visualized using Tanon 5,200 multi System (Tannon, China).

### 2.10 Measurement of intracellular labile iron pool (LIP)

Intracellular LIP was measured by BioTracker 575 Red Fe^2+^ Dye (Sigma-Aldrich, United States; also named FeRhoNox^™^-1), an activatable fluorescent probe that specifically detects labile ferrous ion in living cells (Niwa et al., 2014). In brief, firstly, the cells were exposed to 5 *μ*M FeRhoNox^™^-1 for 37°C, 5% CO_2_ for 30 min after twice PBS washing. Then rinsed the cells with PBS three times and observed cells by fluorescence inverted microscope Axio Observer D1 (ZEISS, Germany). The stained cells were quantified by the flow cytometer BD FACSCanto II (BD Biosciences, United States).

### 2.11 Drug combination test and synergy analysis

HeLa and SiHa cells were treated with different concentrations of DOX (0, 0.1, 0.2, 0.5, 1 and 2 *μ*M) with or without DHA (0, 5, 10, 20 and 40 *μ*M). After 48 h of treatment, the cell viability of HeLa and SiHa cells was measured. The online SynergyFinder software (https://synergyfinder.fimm.fi) was used to calculate drug synergy scoring by four separate reference models (Zero Interaction Potency (ZIP) model, Bliss Independence model, Loewe Additivity model, and Highest Single Agent (HSA) model) (Ianevski et al., 2022). Based on these reference models, if 3 or more models agreed, the combination was synergistic. And the synergy score value > 10 is considered synergistic, between −10 and +10 is considered additive, and a synergy score <−10 is considered antagonistic (Neal et al., 2021).

### 2.12 Statistical analysis

Data analysis was performed using SPSS 24.0. All experimental data were represented as mean ± standard deviation, and the differences between which were analyzed for significance using independent sample *t*-test or one-way analysis of variance (ANOVA) for multivariate analysis. Differences with *p* < 0.05 were deemed statistically significant.

## 3 Results

### 3.1 DHA inhibits the proliferation of cervical cancer cells by inducing ROS

**FIGURE 1:**
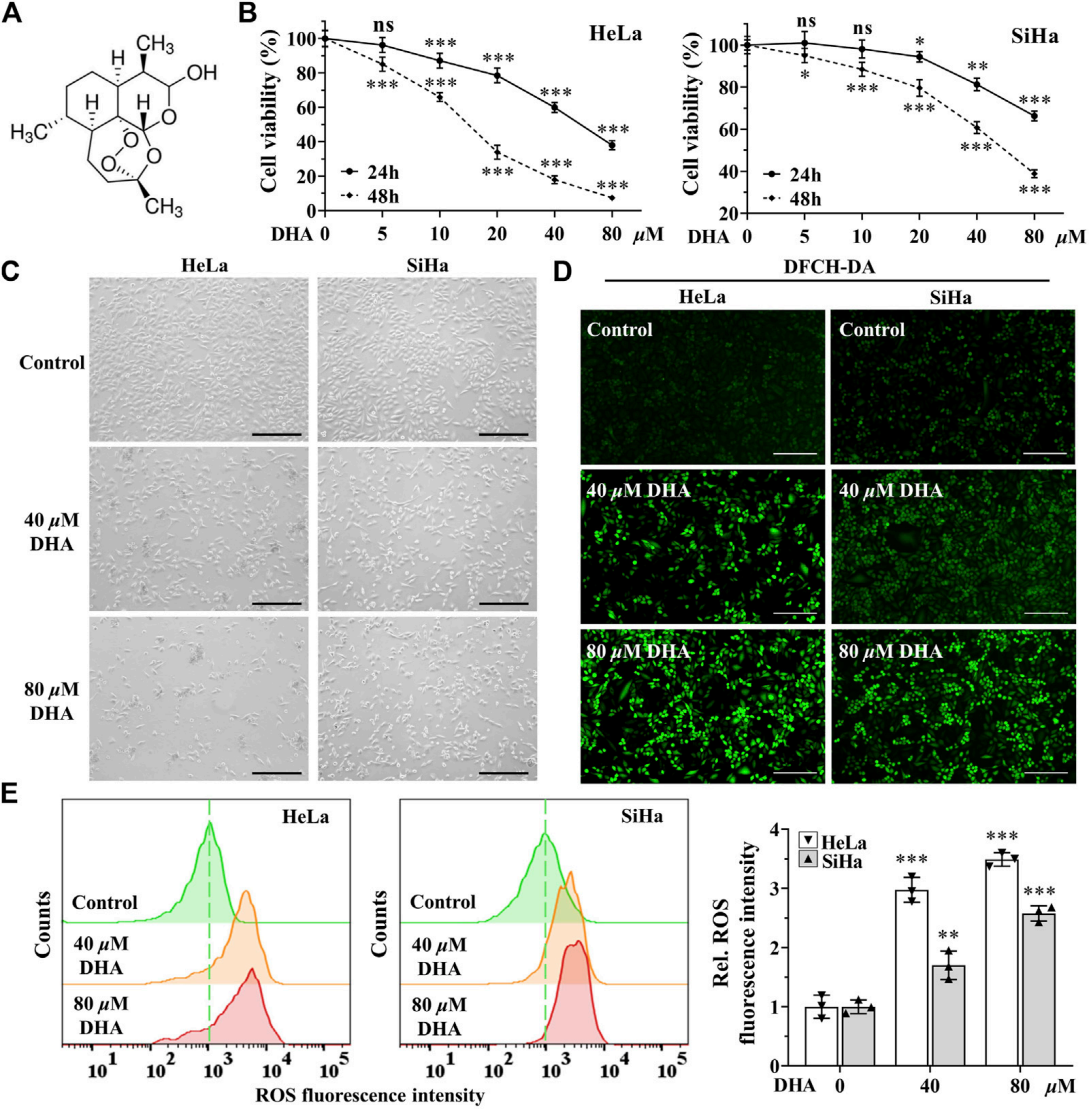
DHA inhibits the proliferation of cervical cancer cells by inducing ROS. **(A)** DHA structure. **(B)** Effects of various concentrations of DHA for 24 or 48 h on the cell viability of HeLa and SiHa cells. **(C)** Morphological changes of HeLa and SiHa cells after 24 h DHA treatment (100×; Scale bar: 200 *µ*m). **(D)** The images and **(E)** fluorescence intensity of intracellular ROS stained by DCFH-DA (100×; Scale bar: 200 *µ*m). DHA: dihydroartemisinin; Rel. relative; ROS: reactive oxygen species. (*, *p* < 0.05; **, *p* < 0.01; ***, *p* < 0.001 compared with control group; ns: no significance.).

DHA is an endoperoxide sesquiterpene lactone and can generate cytotoxic radical species *via* cleavage of the endoperoxide bond (Figure 1A). To investigate the effect of DHA on the proliferation of cervical cancer cells and to select the appropriate intervening time and concentration, cytotoxicity assays were performed with CCK-8 kit. As shown in Figure 1B, the cell viability of HeLa and SiHa cells were significantly inhibited by DHA treatment, which showed a concentration-dependent and time-dependent manner. At concentration of 80 *μ*M DHA, HeLa cells were almost completely killed (inhibition reached 95.36%), while SiHa cells survived less than 40% after 48 h of DHA intervention. To further evaluate the effect of DHA on cell death, cell morphology was observed. The results suggested DHA greatly increased the number of dead cells, and its inhibitory effect on HeLa cells was much greater than that of SiHa cells (Figure 1C). Based on these data, we carried out the subsequent experiments with the concentration of 40 and 80 *μ*M to further elaborate the detailed mechanism of DHA. Besides, DCHF-DA staining showed dramatical increase in fluorescence intensity along with the concentration of DHA (Figures 1D,E) in both cervical cancer cells, reflecting intracellular ROS excessive accumulation. These results indicated that the inhibitory effect of DHA on cervical cancer cell proliferation was related to the induction and accumulation of ROS.

### 3.2 DHA enhances oxidative stress in cervical cancer cells

**FIGURE 2:**
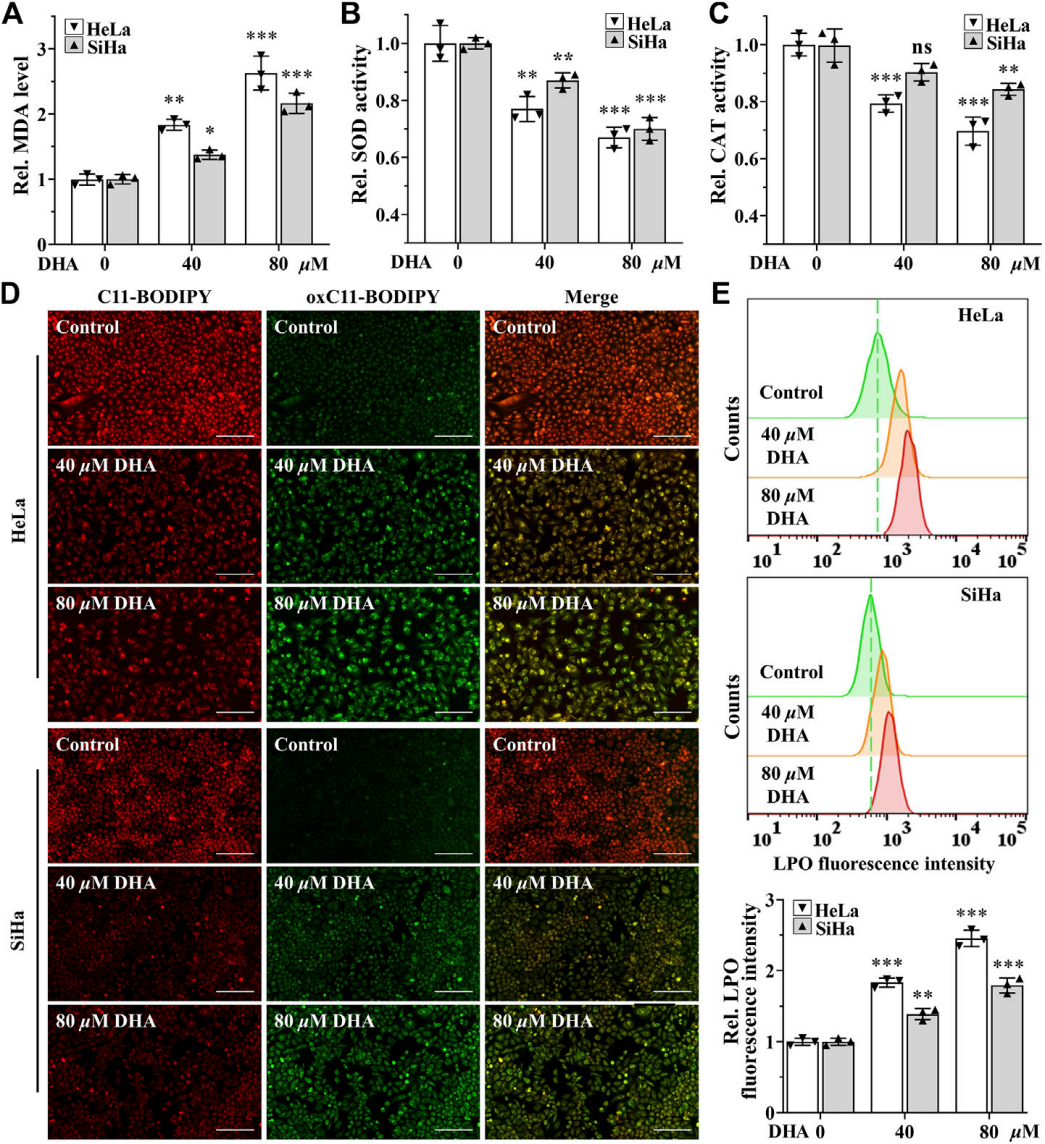
DHA enhances oxidative stress in cervical cancer cells. Levels of **(A)** MDA, **(B)** SOD and **(C)** CAT in HeLa and SiHa cells treated with 0, 40 and 80 *μ*M DHA for 24 h. **(D)** The images and **(E)** fluorescence intensity of intracellular LPO stained by BODIPY^™^ 581/591 C11 (100×; Scale bar: 200 *µ*m). C11-BODIPY represents the level of staining with the probe (unoxidized), while oxC11-BODIPY (oxidized) represents the level of LPO. DHA: dihydroartemisinin; Rel. relative; MDA: malondialdehyde; SOD: superoxide dismutase; CAT: catalase; LPO: liquid peroxidation. (*, *p* < 0.05; **, *p* < 0.01; ***, *p* < 0.001 compared with control group; ns: no significance.).

Higher levels of ROS have been found to promote anticancer signaling by initiating oxidative stress-induced cancer cell death (Arfin et al., 2021). To determine the effect of DHA on oxidative stress in cervical cancer cells, the indexes of oxidative stress including MDA, SOD, CAT and LPO were examined. First, after 24 h of DHA intervention, MDA, one of the most important products of membrane lipid peroxidation, was also prominently increased in both cervical cancer cells (Figure 2A). On the contrary, SOD and CAT activities, two kinds of most important antioxidants, were significantly decreased in DHA groups of HeLa and SiHa cells and the ratios of decrease were positively proportional to the concentration (Figures 2B,C). Next, the changes of LPO after DHA intervention were detected with the specific fluorescent probe BODIPY^™^ 581/591 C11. C11-BODIPY staining showed that the levels of oxC11-BODIPY (oxidized) rose significantly in DHA groups compared with control group in HeLa and SiHa cells (Figures 2D,E), representing DHA exacerbated LPO levels. These assays suggested that DHA enhanced oxidative stress in cervical cancer cells.

### 3.3 DHA triggers cervical cancer cells ferroptosis

**FIGURE 3:**
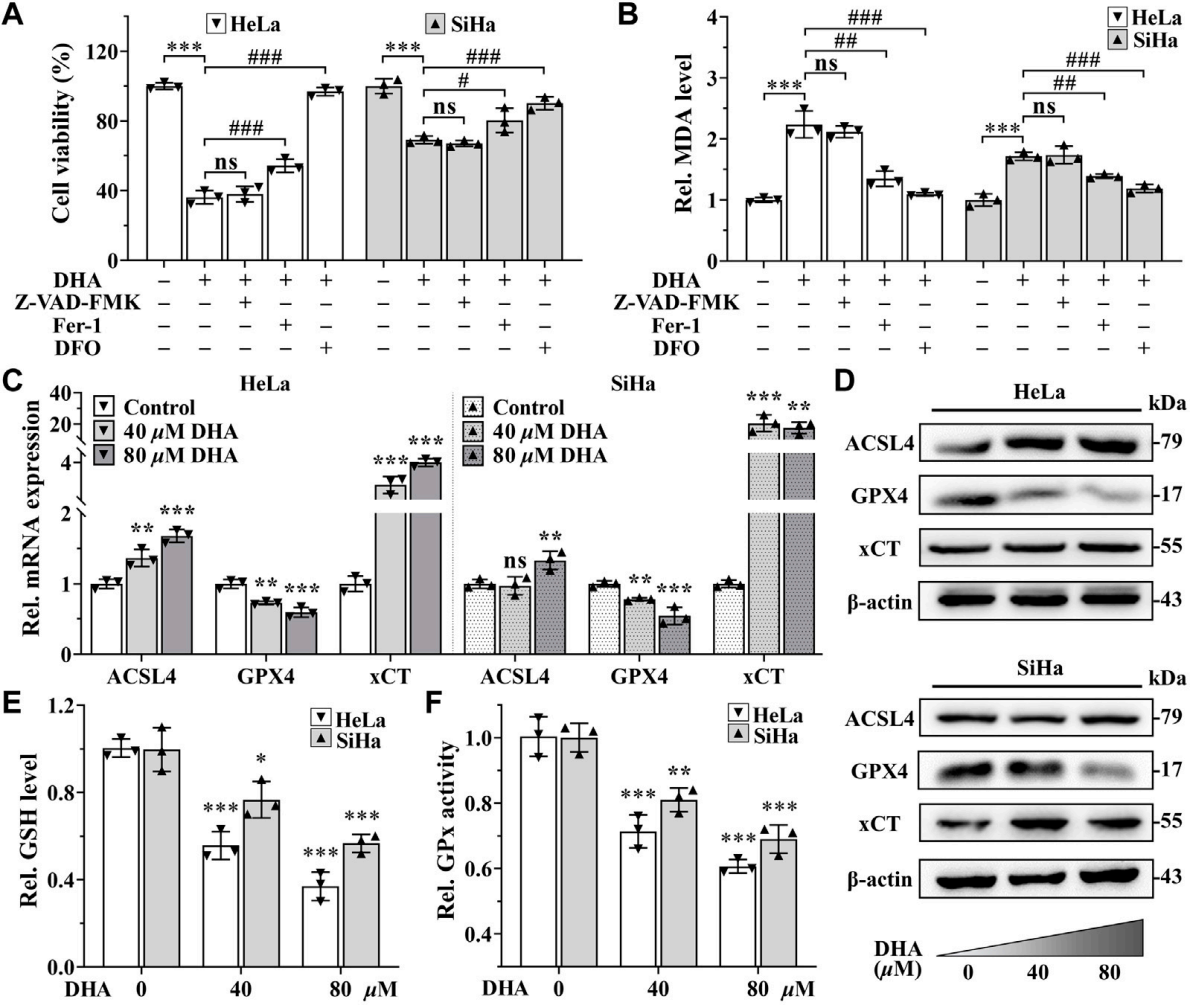
DHA triggers cervical cancer cells ferroptosis. Effects on **(A)** cell viability and **(B)** MDA level of HeLa and SiHa cells after 24 h of DHA treatment with or without Z-VAD-FMK, Fer-1 or DFO. **(C)** The transcriptional levels of *ACSL4*, *GPX4* and *xCT* after DHA treatment. **(D)** The protein expressions of ACSL4, GPX4 and xCT after DHA treatment, compared with β-actin. Levels of **(E)** GSH and **(F)** GPx in HeLa and SiHa cells treated with 0, 40 and 80 *μ*M DHA for 24 h. Rel. relative; DHA: dihydroartemisinin; MDA: malondialdehyde; Fer-1: ferrostatin-1; DFO: deferoxamine; ACSL4: acyl-CoA synthetase long-chain family member 4; GPX4: glutathione peroxidase 4; xCT: cystine-glutamate antiporter; GSH: glutathione; GPx: glutathione peroxidase. (*, *p* < 0.05; **, *p* < 0.01; ***, *p* < 0.001 compared with control group; #, *p* < 0.05; ##, *p* < 0.01; ###, *p* < 0.001 compared with DHA group; ns: no significance.).

Excessive accumulation of LPO is one of the characteristic features of ferroptosis (Dixon et al., 2012). To further determine whether ferroptosis contributed to the cell death induced by DHA, apoptosis inhibitor Z-VAD-FMK (50 *μ*M), ferroptosis inhibitors Fer-1 (5 *μ*M) or iron chelator DFO (50 *μ*M) was co-treated with 80 *μ*M DHA for 24 h. From Figure 3A, it was obvious that the inhibition effect induced by DHA was alleviated by the addition of Fer-1, and even completely reversed in adding DFO group, while Z-VAD-FMK could not reverse DHA-induced cell death in HeLa and SiHa cells. Correspondingly, ferroptosis inhibitors also significantly reduced the high level of MDA induced by DHA, but apoptosis inhibitor had no effect (Figure 3B). These results indicated that ferroptosis may as the central method that contributed to DHA-caused cell death.

To further verify the occurrence of ferroptosis, next we detected the expression of ferroptosis related genes. The detection revealed that, compared with control group, the mRNA and protein expression of gene *GPX4* were both downregulated in a concentration-dependent manner after 24 h of DHA treatment in both cell lines (Figures 3C,D), while the expression of gene *Acyl-CoA synthetase long-chain family member 4 (ACSL4)* were opposite (Figures 3C,D). In SiHa cells, the transcriptional and protein levels of cystine-glutamate antiporter (xCT) gene were both significantly elevated after DHA intervention, even the highest upregulated in mRNA level more than 20 times (Figures 3C,D). Although *xCT* was also highly expressed at mRNA level in HeLa cells (Figure 3C), the protein change was inconspicuous (Figure 3D). In the meantime, GSH and GPx, which exert anti-ferroptosis effects, also decreased after DHA treatment in a concentration-dependent manner in both cell lines (Figures 3E,F). Altogether, these results confirmed that DHA triggered cervical cancer cells ferroptosis, which was related to GPX4 depletion.

### 3.4 DHA induces ferritinophagy-dependent ferroptosis in cervical cancer cells

**FIGURE 4:**
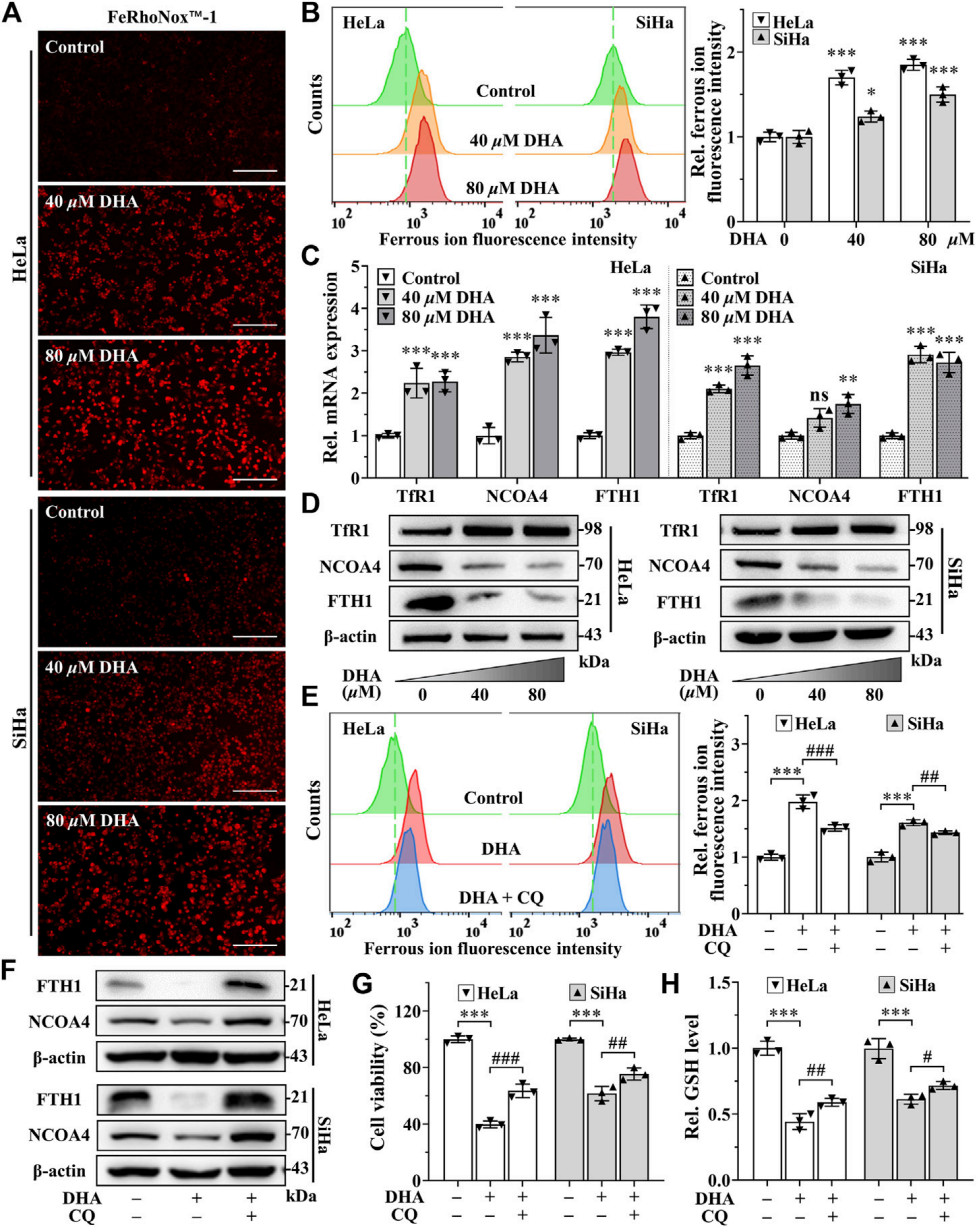
DHA induces ferritinophagy-dependent ferroptosis in cervical cancer cells. **(A)** The images and **(B)** fluorescence intensity of intracellular ferrous ion stained by FeRhoNox™-1 (100×; Scale bar: 200 *µ*m). **(C)** The transcriptional levels of *TfR1*, *NCOA4* and *FTH1* after DHA treatment. **(D)** The protein expressions of TfR1, NCOA4 and FTH1 after DHA treatment, compared with β-actin. Effects on **(E)** intracellular ferrous ion level, **(F)** FTH1 and NCOA4 protein expressions, **(G)** cell viability and **(H)** GSH level of HeLa and SiHa cells after 24 h of DHA treatment with or without CQ. DHA: dihydroartemisinin; Rel. relative; TfR1: transferrin receptor 1; NCOA4: nuclear receptor coactivator 4; FTH1: ferritin heavy chain 1; CQ: chloroquine; GSH: glutathione. (*, *p* < 0.05; **, *p* < 0.01; ***, *p* < 0.001 compared with control group; #, *p* < 0.05; ##, *p* < 0.01; ###, *p* < 0.001 compared with DHA group; ns: no significance.).

Since iron is an important part of ferroptosis (Stockwell, 2022), and combined with previous results that the iron chelator DFO was more efficient than Fer-1 in inhibiting DHA-induced cell death, we speculated that iron may play a momentous role in DHA-induced ferroptosis. So, the impact of DHA treatment on intracellular LIP was evaluated. As shown in Figures 4A,B, the fluorescence intensities of DHA groups reinforced remarkably compared with control group in cervical cancer cells, suggesting that DHA significantly increased the levels of LIP, which could promote cell ferroptosis (Lu et al., 2021).

Then, we further examined the effect of DHA on iron metabolism. The data from qPCR showed that DHA intervention elevated the mRNA expression of *transferrin receptor 1* (*TFR1*), *NCOA4* and *ferritin heavy chain 1* (*FTH1*) in both cell lines (Figure 4C). And at the protein level, the trend of TfR1 was consistent with mRNA (Figure 4D). However, the protein expression of FTH1 and NCOA4 displayed the opposite trend with mRNA (Figure 4D). It is noteworthy that FTH1 degradation exhibited a synergistic effect with NCOA4 depletion according to Western blotting results (Figure 4D), which may be caused by the delivery of ferritin by NCOA4 to lysosomes and subsequent the ferritinophagy occurred. Since the ferritin degradation was *via* autophagy, autophagy inhibitor CQ (25 *μ*M) was co-cultured with 80 *μ*M DHA for 24 h to verify whether autophagy contributes to DHA-induced FTH1 degradation and ferroptosis. As expected, CQ addition could significantly inhibit the degradation of FTH1 and the depletion of NCOA4 (Figure 4F), and reduce LIP levels (Figure 4E). At the same time, the inhibitory effect of DHA on HeLa and SiHa cells also alleviated by CQ (Figure 4G), accompanied by a decrease in GSH consumption (Figure 4H). All these data indicated that ferritinophagy-dependent ferroptosis contribute to DHA-induced cell death.

### 3.5 HO-1 against DHA-induced ferroptosis in cervical cancer cells

**FIGURE 5:**
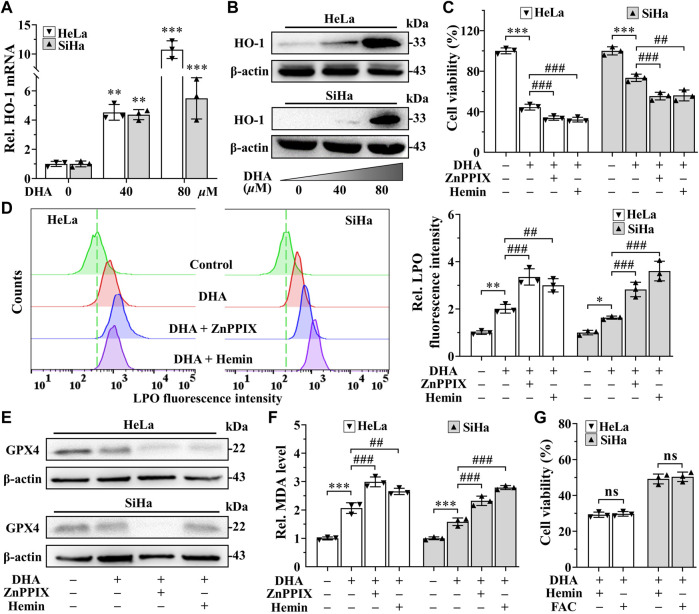
HO-1 against DHA-induced ferroptosis in cervical cancer cells. **(A)** The transcriptional level and **(B)** protein expression of HO-1 of HeLa and SiHa cells after 24 h of treatment with DHA. Effects on **(C)** cell viability, **(D)** LPO level, **(E)** GPX4 protein expression and **(F)** MDA level of HeLa and SiHa cells after 24 h of DHA treatment with or without ZnPPIX or hemin. **(G)** The cell viability of HeLa and SiHa cells treated with DHA supplemented Hemin or FAC. Rel. relative; DHA: dihydroartemisinin; HO-1: heme oxygenase-1; ZnPPIX: protoporphyrin IX zinc(II); LPO: liquid peroxidation; GPX4: glutathione peroxidase 4; MDA: malondialdehyde. (*, *p* < 0.05; **, *p* < 0.01; ***, *p* < 0.001 compared with control group; #, *p* < 0.05; ##, *p* < 0.01; ###, *p* < 0.001 compared with DHA group; ns: no significance.).

In addition, the extremely high expression of heme oxygenase-1 (HO-1) after DHA treatment, especially in 80 *μ*M DHA group (Figures 5A,B), attracted our attention. Considering the dual function of HO-1 (Ryter, 2021), its inhibitor ZnPPIX and agonists hemin were used to investigate its role in DHA-induced ferroptosis. Confusingly, incubation of DHA with either ZnPPIX (10 *μ*M) or hemin (25 *μ*M) both significantly increased the cell growth inhibition rates in cervical cancer cells (Figure 5C), along with higher levels of LPO and MDA (Figures 5D,F). And the protein level of GPX4 were further suppressed in both combination groups (Figure 5E). Given the critical role of iron in ferroptosis, we surmised that the boosting anticancer action of ZnPPIX with DHA should be through inhibition of HO-1, while the unexpected performance of hemin may not be related to HO-1 but mediated by iron. To address this question, FAC, a commonly used mono-iron compound, was chosen as a control to explore the effect of iron on DHA. At the same concentrations, we compared the consequences of incubating DHA with hemin or FAC. As Figure 5G shown, the cell viability showed no significant difference between DHA + hemin group and DHA + FAC group in both cell lines, hinting that iron rather than HO-1 was the dominant factor for hemin to increase DHA inhibitory effect on cervical cancer cells. Thus, these results suggested that HO-1 may play a role in resisting ferroptosis in DHA-induced cervical cancer cell death.

### 3.6 DHA sensitizes cervical cancer to DOX-induced cell death

**FIGURE 6:**
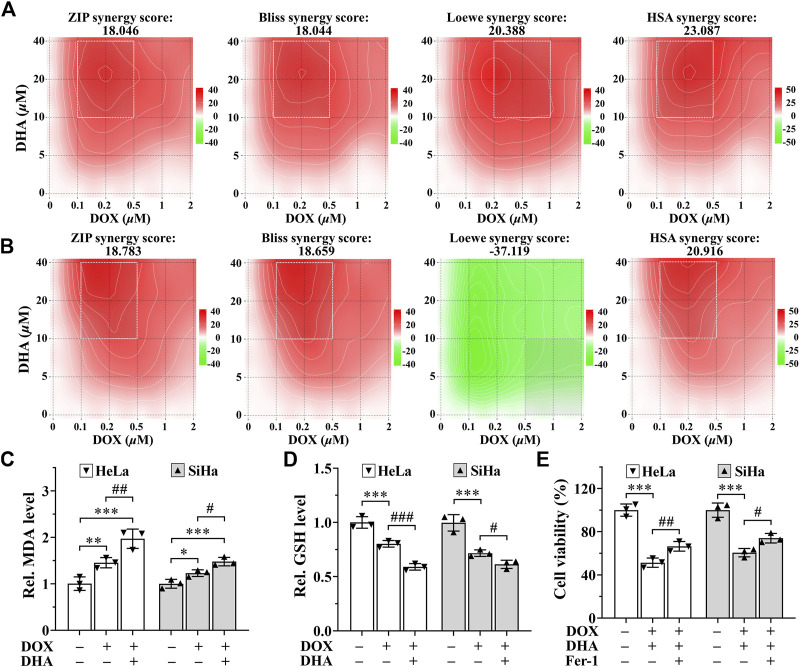
DHA sensitizes cervical cancer to DOX-induced cell death. **(A, B)** Heatmaps of drug combination responses. Synergy score >10 is considered synergistic, between −10 and +10 is considered additive, <−10 is considered antagonistic. The gradation of the red regions indicates the intensity of synergism. The white rectangle indicates the concentrations encompassing the region of highest synergy. Effects on the levels of **(C)** MDA and **(D)** GSH in HeLa and SiHa cells after 24 h of treatment with DOX with or without DHA. **(E)** Effects of Fer-1 on cell viability of HeLa and SiHa cells treated with DOX and DHA. Rel. relative; DHA: dihydroartemisinin; DOX: doxorubicin; MDA: malondialdehyde; GSH: glutathione; Fer-1: ferrostatin-1. (*, *p* < 0.05; **, *p* < 0.01; ***, *p* < 0.001 compared with control group; #, *p* < 0.05; ##, *p* < 0.01; ###, *p* < 0.001 compared with DOX group).

SynergyFinder is a web-application for interactive analysis and visualization of multi-drug combination response data (Ianevski et al., 2022). In HeLa cells, synergy scoring of treatment with DHA and DOX calculated by four separate reference models were all >10, which was considered strongly synergistic between DHA and DOX. And except Loewe Additivity model, the other three reference models all revealed highly synergistic effect between DHA and DOX in inhibiting SiHa cell proliferation (synergy scores >10). Under the previous rule, we also expected this to be synergistic. As shown in Figures 6A,B, the white rectangle indicates the concentrations encompassing the region of the maximum synergistic area. The data indicated that 10 *μ*M for DHA and 0.2 *μ*M for DOX were the lowest concentrations encompassing the region of highest synergy, which were selected as the best-combined concentration for DHA and DOX. Therefore, this synergistic combination of DHA and DOX was used in our following experiments. After 24 h of treatment, it could be seen that, compared with DOX group, the level of MDA in DHA + DOX group of HeLa and SiHa cells were increased obviously, while the level of GSH were significantly lower (Figures 6C,D). In addition, the inhibition effect induced by DHA + DOX could also be alleviated by Fer-1 (Figure 6E). The results indicated that the synergistic lethal effect of DHA with DOX in cervical cancer was also related to ferroptosis.

## 4 Discussion

Cervical cancer is still one of the most common and lethal gynecological malignancy and the clinical therapeutics is limited due to the drug resistance and metastasis of tumor, seriously threating women health (Arbyn et al., 2020; Sung et al., 2021). Therefore, it is imperative and prevalent to explore more efficient and safer therapeutic targets and therapies. As mentioned previously, cancer cells are more susceptible to ferroptosis (Chen et al., 2022). The most likely reason to explain this phenomenon is that the rapid growth and metabolism lead to the intense iron demand (Torti and Torti, 2013) and high levels of intracellular ROS (Dixon and Stockwell, 2014) in cancer cells. Furthermore, encouragingly, researchers have found that the cancer cells with resistance to apoptosis and chemotherapy are also exquisitely susceptible to ferroptosis (Hangauer et al., 2017; Tsoi et al., 2018), which further emphasize the prospect of ferroptosis as a novel target for antitumor therapy. In addition, NCOA4-mediated ferritinophagy can enhance ferroptosis by inducing the degradation of ferritin and increasing LIP (Zhou et al., 2020; Yu et al., 2022). In recent years, the role of ferroptosis in cervical carcinogenesis, progression and immunity has been gradually concerned (Qi et al., 2021; Yang et al., 2022). Except sorafenib, natural product oleanolic acid inhibited cervical cancer Hela cell proliferation through modulation of the ACSL4 ferroptosis signaling pathway (Xiaofei et al., 2021). As a natural ferroptosis inducer, DHA could react with ferrous ions to produce cytotoxic ROS and played an important role in inducing ferroptosis (Lin et al., 2016; Shen et al., 2020). And in the present study, we reveal the molecular mechanisms that DHA triggered ferritinophagy-dependent ferroptosis in cervical cancer and sensitized to DOX, which may provide novel avenues for future therapy development.

Since structural diversity and biological prevalidation, natural products are indispensable sources of clinical drug research and development (Hong et al., 2020; Kim et al., 2021). In the field of cancer therapy, natural products also show potential anticancer effects, and its use has facilitated the development of effective and safer anti-cancer drugs. Currently, a large number of studies have been reported on natural products to treat cancer and overcome tumor drug resistance (Zhang et al., 2020b; Dahmardeh Ghalehno et al., 2022). DHA, a natural anticancer drug, has exhibited a variety of anticancer properties such as inducing apoptosis or autophagy and even can reverse drug resistance of certain cancer cell lines and greatly enhance the anticancer effect in combination with a variety of chemotherapeutic drugs (Dai et al., 2021). Moreover, no obvious toxicity in normal cells has been found after DHA treatment, indicating that DHA is a potential ideal anti-cancer drug (Li et al., 2021). With the deepening of research, its role in inducing ferroptosis was gradually discovered (Hong et al., 2020; Kim et al., 2021). For cervical cancer cells, it is confirmed that DHA has a potent lethal effect and synergistic effect with chemotherapeutic drugs (Tai et al., 2016; Tang et al., 2021b), while the self-assembled DHA nanoparticles are a highly promising delivery system for targeted cancer treatment (Lu et al., 2020). However, it remains rarely explored and unclear in cervical cancer about the roles of DHA for ferroptosis.

**FIGURE 7:**
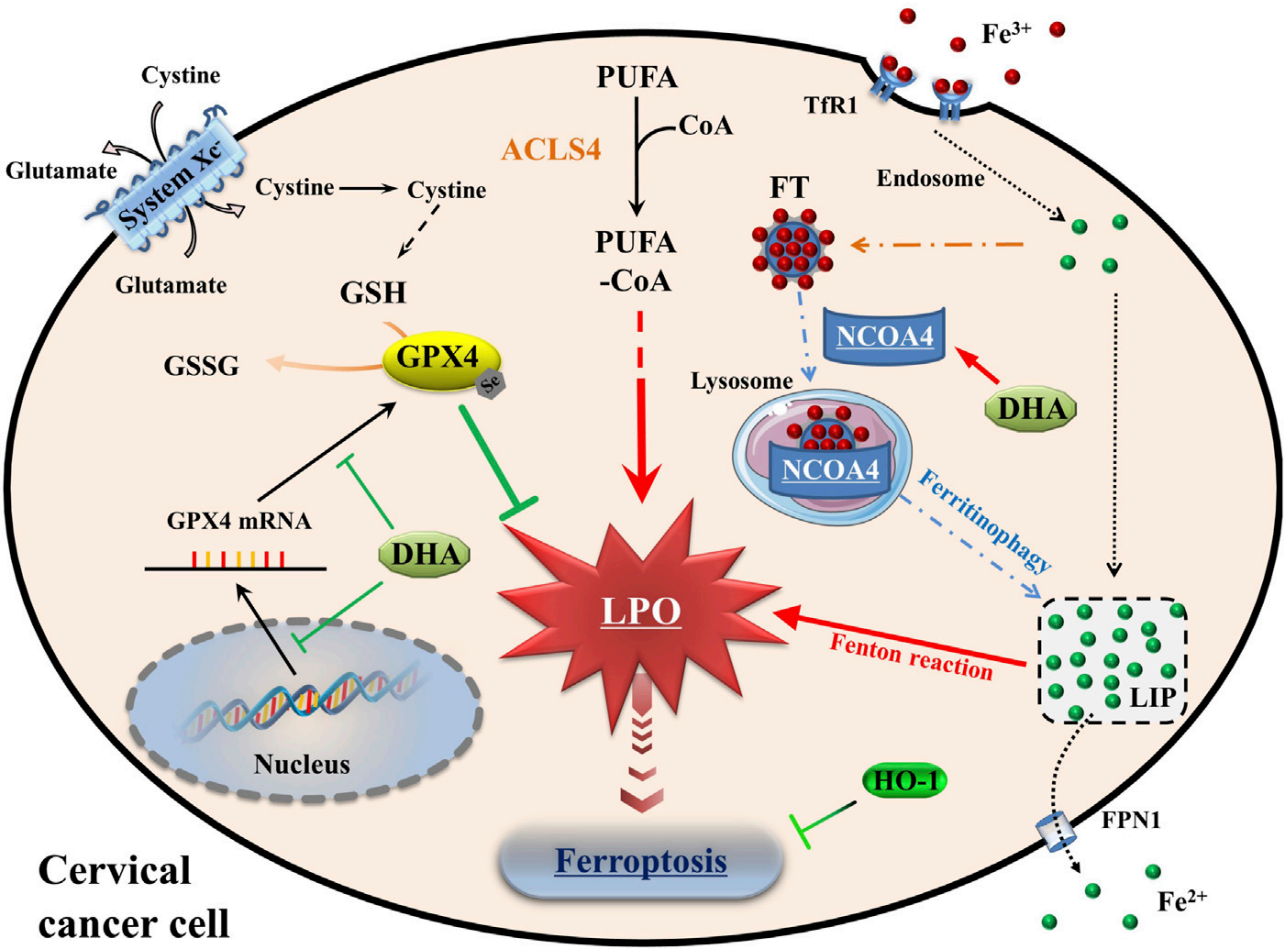
Schematic depicting DHA-induced ferroptosis in cervical cancer cells. DHA: dihydroartemisinin; GPX4: glutathione peroxidase 4; GSH: glutathione; GSSG: oxidized glutathione; CoA: coenzyme A; PUFA: polyunsaturated fatty acids; ACSL4: acyl-CoA synthetase long-chain family member 4; PLO: liquid peroxidation; Fe^3+^, ferric iron; Fe^2+^, ferrous iron; TfR1: transferrin receptor 1; Steap3: six transmembrane epithelial antigen of the prostate 3; FT: ferritin; NCOA4: nuclear receptor coactivator 4; LIP: labile iron pool; FPN1: Ferroportin1; HO-1: heme oxygenase-1.

At the cellular level, the pathways of iron, amino acids and lipid metabolism are involved in the initiation and execution of ferroptosis (Du et al., 2022). As Figure 7 shown, iron, transported into cells by TfR1, is either stored in ferritin or exported by FPN1. The excess cellular iron, particularly ferrous iron, can react directly with cellular oxidants to produce cytotoxic hydroxyl radicals *via* the Fenton reaction, which in turn promotes ferroptosis (Dixon and Stockwell, 2014). And the key enzyme of lipid peroxidation ACSL4 plays a role in responsible for the esterification of coenzyme A to polyunsaturated fatty acids (Killion et al., 2018). It is considered to be an essential component for ferroptosis execution, as its renders the cell more susceptible to ferroptosis (Doll et al., 2017). GPX4 is the only known GPX that can catalyze toxic lipid hydroperoxides into non-toxic lipid alcohols under normal physiological conditions, with its substrate GSH (Ingold et al., 2018). With the System Xc^−^, GPX4 constitutes the main cellular pathway to protect cells from undergoing ferroptosis.

Oxidative stress is caused by an imbalance between cellular oxidants and antioxidants (Arfin et al., 2021). When ferroptosis occurs, cellular oxidative stress is intensified. So, the oxidative stress levels were assessed firstly to understand the cellular state. In cervical cancer cells, DHA treatment aggravated the levels of oxidative stress, which was manifested by the accumulation of ROS, LPO and MDA, and the upregulation of ACSL4. And the cell death of cervical cancer cells caused by DHA, accompanied with depletion of GPX4 and GSH, could be attenuated by ferroptosis inhibitors. In accordance with these data, we concluded that DHA triggered ferroptosis, which was related to GPX4 depletion. Likewise, in glioblastoma and lung cancer cells, DHA induced ferroptosis by inhibiting xCT/GPX4 axis in different ways (Yi et al., 2020; Yuan et al., 2020), indicating that the mechanisms of DHA-induced ferroptosis in various cells were different. Simultaneously, in HeLa and SiHa cells, the synergistic lethal effect between DHA and DOX, used as a first-line drug to treat cervical cancer (Wang et al., 2022), was related to ferroptosis. This suggested that DHA may have potential as an adjunct to chemotherapy.

Besides, Fenton reaction, caused by the interaction between excessive ferrous iron and peroxide leading to the production of highly active hydroxyl radicals (Dixon and Stockwell, 2014), was also a predisposing factor of ferroptosis. Notably, many cellular processes change the sensitivity of cells to ferroptosis by altering cellular LIP levels (Xiao et al., 2020; Lu et al., 2021). Among them, ferritinophagy mediated by selective cargo receptor NCOA4 is a major pathway to regulate intracellular LIP levels, which delivers ferritin to lysosomes *via* macroautophagy to release stored iron for cellular utilization (Mancias et al., 2014). On one hand, due to the siderophilic properties of cancer, NCOA4-mediated ferritinophagy may promote the progression of some tumors (Santana-Codina et al., 2022). On the other hand, excessive LIP accumulation by ferritinophagy could initiate ferritinophagy-dependent ferroptosis and played an anticancer role (Wang et al., 2021b; Li et al., 2022b; Stockwell, 2022). As mentioned previously, the degradation of ferritin induced by DHA was an important consideration leading to ferroptosis (Du et al., 2019; Chen et al., 2020). Indeed, the significant degradation of FTH1 and subsequent increases of intracellular ferrous iron were observed in our study, which may be due to the increased efficiency of NCOA4-mediated ferritinophagy caused by DHA. Thus, DHA-induced ferritinophagy may be one of the causes of initiating and enhancing ferroptosis in cervical cancer cells. However, the specific mechanism remains to be further studied.

As we known, HO-1 is an oxidative stress inducing enzyme that catalyzes the degradation of heme into biliverdin, carbon monoxide and ferrous iron. Given the dual role of HO-1 in regulating iron and ROS homeostasis, its contradictory role in ferroptosis may depend on the degree of ROS production and the following oxidative damage (Ryter, 2021). Gloria et al. found that Siramesine and Lapatinib induced a synergistic ferroptosis through reduced HO-1 Levels (Villalpando-Rodriguez et al., 2019). However, luteolin, a natural compound monomer, triggered ferroptosis in clear cell renal cell carcinoma by excessively up-regulating HO-1 expression and activating LIP (Han et al., 2022). Currently, pharmacological and genetic tools have proposed cancer therapy strategies of targeting HO-1 (Chiang et al., 2018). The extremely high expression of HO-1 induced by DHA was seen in HeLa and SiHa cells, which was similar to the result in glioblastoma and was worthy of further research (Yi et al., 2020). After using specific HO-1 inhibitor ZnPPIX, the degree of DHA-induced ferroptosis was aggravated. So, another important conclusion of our work is the fact that HO-1 exerted antioxidant effects against DHA-induced ferroptosis. On the other hand, it had been reported that DOX could downregulate Nrf2 to inhibit HO-1 and GPX4 levels (Li et al., 2022a), which may be one of the important reasons for its synergism with DHA to induce ferroptosis. The combination of DHA and HO-1 inhibitors may have a potential application in cancer therapy by mediating the induction of ferroptosis.

However, there are several limitations to our work. Due to limited resources, our research was conducted only in cell lines without animal experiments. The mechanisms of ferroptosis are delicate and complicated. In addition to the metabolism of iron, amino acids and lipid, mitochondria also act a critical role in regulating ferroptosis (Takashi et al., 2020), which are not included in this study. Hence, further evaluations of the specific molecular mechanism underlying DHA-mediated regulation of ferroptosis and its effect on upstream pathway-related proteins is needed. In the next step, future studies will focus on these deficiencies and carry out more in-depth research.

## 5 Conclusion

Taken together, our study demonstrated evidence that the inhibitory effect of DHA on the proliferation of cervical cancer is related to ferroptosis, mediated by the GPX4 inhibition and ferritinophagy, whereas HO-1 expression is anti-ferroptosis. Furthermore, the synergistic lethal effect of DHA with chemotherapeutic agents makes it possible to be a potential adjuvant drug for chemotherapy. All these findings paved the way for further research and provided the theoretical basis for its clinical application.

## Data Availability

The original contributions presented in the study are included in the article/supplementary material, further inquiries can be directed to the corresponding author.
